# Noise Exposure and Hearing Impairment among Chinese Restaurant Workers and Entertainment Employees in Hong Kong

**DOI:** 10.1371/journal.pone.0070674

**Published:** 2013-08-15

**Authors:** Xiang Qian Lao, Ignatius Tak Sun Yu, Dennis Kin Kwok Au, Yuk Lan Chiu, Claudie Chiu Yi Wong, Tze Wai Wong

**Affiliations:** 1 School of Public Health and Primary Care, the Chinese University of Hong Kong, Hong Kong, People's Republic of China; 2 Department of Surgery, the University of Hong Kong, Hong Kong, People's Republic of China; University of Cincinnati, United States of America

## Abstract

**Background:**

Noise-induced hearing loss (NIHL) is a major concern in the non-manufacturing industries. This study aimed to investigate the occupational noise exposure and the NIHL among Chinese restaurant workers and entertainment employees working in the service industry in Hong Kong.

**Methods:**

This cross-sectional survey involved a total of 1,670 participants. Among them, 937 were randomly selected from the workers of Chinese restaurants and 733 were selected from workers in three entertainment sectors: radio and television stations; cultural performance halls or auditoria of the Leisure and Cultural Services Department (LCSD); and karaoke bars. Noise exposure levels were measured in the sampled restaurants and entertainment sectors. Each participant received an audiometric screening test. Those who were found to have abnormalities were required to take another diagnostic test in the health center. The “Klockhoff digit” method was used to classify NIHL in the present study.

**Results:**

The main source of noise inside restaurants was the stoves. The mean hearing thresholds showed a typical dip at 3 to 6 KHz and a substantial proportion (23.7%) of the workers fulfilled the criteria for presumptive NIHL. For entertainment sectors, employees in radio and television stations generally had higher exposure levels than those in the halls or auditoria of the LCSD and karaoke bars. The mean hearing thresholds showed a typical dip at 6 KHz and a substantial proportion of the employees fulfilled the criteria for presumptive NIHL (38.6%, 95%CI: 35.1–42.1%). Being male, older, and having longer service and daily alcohol consumption were associated with noise-induced hearing impairment both in restaurant workers and entertainment employees.

**Conclusion:**

Excessive noise exposure is common in the Chinese restaurant and entertainment industries and a substantial proportion of restaurant workers and entertainment employees suffer from NIHL. Comprehensive hearing conservation programs should be introduced to the service industry in Hong Kong.

## Introduction

Occupational NIHL has been regarded as a global public health problem, and systematic research on occupational NIHL was performed as early as the late 19th century [1]. It has been estimated that around nine million workers in the USA are exposed to a time-weighted average (TWA) sound level of 85 dBA or above [2]. In Europe, a survey has shown that 28% of workers are exposed to noise levels of approximately 85–90 dBA [3]. About 16% of hearing loss worldwide is attributable to occupational noise exposure [4], [5]. During the last few decades, most occupational NIHL researches and preventive strategies have been focused mainly on the workers in traditional industries. With the recent shift in the economy from a manufacturing base to a service base, there has been growing concern that NIHL affects not only the traditional noisy trades, but also many employees in the non-manufacturing industries such as the service industry.

However, few studies have been carried out to ascertain the size of the exposed group and the magnitude of the problem in the service sector. Employees who work in Chinese restaurants in Hong Kong are an obvious target group, because noise-generating pressurized gas stoves are frequently used in Chinese restaurants for Chinese-style cooking. At present, little data on noise in Chinese restaurants are available. A systematic and scientific evaluation of noise levels in Chinese restaurants and the collection of baseline prevalence data on the hearing levels of the workers are essential for developing occupational health control measures.

In addition to restaurant workers, many studies have shown that there is a risk of hearing loss among musicians and teenagers who listen to loud music [6]. Employees who work in the entertainment industry are another group at high risk of hearing impairment because of their exposure to loud music while working at nightclubs, pop and rock concerts, and radio and television stations. A small number of studies have been carried out on the employees of nightclubs, and the results have shown that the noise levels in nightclubs ranged from 94.9–106.7 dBA. These studies show that a substantial proportion of the employees suffer from hearing loss [7]–[9]. We have therefore conducted a survey to assess occupational noise exposure and hearing impairment among restaurant workers and entertainment employees in Hong Kong.

## Methods

### Setting, participants and noise assessment

#### (1) Chinese restaurant workers

The Hong Kong Standard Industrial Classifications code for Chinese restaurants is 641 [1], so a list of 1,435 restaurants in the industrial sub-sector 641 was obtained from the Census and Statistics Department. For operational reasons, 29 restaurants with fewer than 25 employees were excluded. To get a representative sample of subjects, the restaurants were selected randomly from the following three size categories of employment: (1) small size (26–50 employees); (2) medium size (51–100 employees); and (3) large size (>100 employees). The number of restaurants to be surveyed under each size category was based on the distribution of employees in restaurants of different sizes. All employees on duty in each selected restaurant were invited to participate in the survey. The employees in Chinese restaurants were categorized into the following four job groups:

- Service workers: workers in the service areas, including managers, captains, waiters/waitresses, pantry helpers, dim sum sales staff, barbecue counter staff, fish tank caretakers, dish-up, clerks, and accountants
- Cooks: including chief cooks, second cooks, dim sum cooks, and barbecue cooks
- Dishwashers
- Kitchen workers: workers inside the kitchens other than cooks.

Based on an estimated 30% prevalence of noise-induced hearing impairment among the exposed workers [10], it was necessary to examine about 900 workers in order to give a precise estimate of the true prevalence with a 95% confidence interval of +/−3% [11], [12]. Taking the response rate into account, 23 restaurants with 1,339 employees were sampled. Among these employees, about 52% were service workers, 9% were cooks, 29% were kitchen workers, and 10% were dishwashers. A total of 937 employees agreed to participate in this survey, and this gave an overall response rate of 70%. The response rates for service workers, cooks, kitchen workers, and dishwashers were 77.4%, 53.8%, 64.7%, and 60.4%, respectively. The study protocol was reviewed and approved by the Survey and Behavioural Research Ethics Committee of Chinese University of Hong Kong. Signed informed consent was obtained from the participant beforehand.

Noise assessment included measuring the noise levels in selected working environments, and measuring the cumulative personal noise exposures for some participants with job titles including cook and dishwasher, which are a typical position having a high risk of noise exposure in Chinese restaurants.

Environmental noise levels were measured by using precision sound level meters (Rion NL14, Japan) in various locations where the workers were working. The measurements were then integrated to give an A-weighted sound pressure level (LAeq) for the time period of the measurements.

Regarding cumulative personal noise exposure, “B & K” noise dosimeters (Model 4436, Denmark) were used to measure the participants' personal daily noise exposure levels (LEP_d_ values). The computing capability of the dosimeter allowed a complete real-time record of the sound levels which the workers were exposed to during the measurement period to be statistically analyzed. The personal exposure data were then transferred from the dosimeters to a personal notebook computer (Compaq, 430c) and they were then processed by using the software supplied with the dosimeters.

#### (2) Entertainment employees

As the scope in the entertainment industry in Hong Kong is very wide, it was difficult to obtain a representative sample. Therefore, three specific sectors and populations were targeted for this study: (1) radio and television stations; (2) cultural performance halls or auditoria of the LCSD; and (3) karaoke bars. One radio and television station, 11 halls or auditoria of the LCSD and two karaoke bars were selected, and their employees were invited to participate in this study. The response rate in each sector varied. For the radio and television stations, a total of 428 employees were invited and 262 of them agreed to participate (response rate: 61.2%); for the 11 halls or auditoria of the LCSD, 613 employees were invited and 438 of them agreed to participate (response rate: 71.5%); for the two karaoke bars, 100 employees were invited and 33 of them agreed to participate (response rate: 33.0%). A total of 733 participants were therefore included in the analysis. The study protocol was reviewed and approved by the Survey and Behavioural Research Ethics Committee of Chinese University of Hong Kong. Signed informed consent was obtained from the participant beforehand.

Noise assessment referred to the measurements of noise levels for (1) typical radio program presenters who wore headsets during work, and (2) typical participants who did not wear headsets during work.

A special method was used to assess the personal noise exposures for radio program presenters who wore headsets while carrying out their duties. The sound pressure levels were measured by using a precision sound level meter (Rion NL14, Japan) via an artificial ear (CUOI) with the earphones (TDH-39P, Denmark) plugged into the monitor interphase that was parallel in output and subjected to adjustment with the headset worn by the presenters. In other words, we measured the sound levels in the artificial ear, which had the same levels of sound pressure as in the ears of the person who wore the headset.

For participants who did not wear headsets, cumulative personal noise exposures were assessed and methods. The methods and equipments were the same as those used for restaurant cooks and dishwashers, described above.

### Audiometric tests

The audiometric tests consisted of a screening test in the field and a diagnostic test in the hearing test center. The screening test was performed for all participants (including all restaurant workers and entertainment employees). Those who were found to have abnormalities during the screening test were then requested to attend the diagnostic audiometric test.

A standard questionnaire was used for all participants during the audiometric tests to collect information such as personal particulars, current and past occupational noise exposures, noise exposures other than from their occupation and the use of ear protectors. An otoscopic examination of the external ear canals and eardrums was then performed and any abnormalities were noted.

Audiometric screening examinations were carried out in the field. For all participants, pure tone air conduction hearing thresholds at different frequencies were measured manually by the abridged ascending method following a standard procedure in accordance with BS 6655: 1986. The hearing thresholds were measured for both ears at the following octave frequencies: 500, 1,000, 2,000, 3,000, 4,000, 6,000, and 8,000 Hz. A portable audiometer (Interacoustics AD 25 or AD 27, Denmark) with earphones (TDH-39P, Denmark) was used to perform the audiometric screening test. The earphones were equipped with audiocups (Amplivox, England) to enable testing in quiet rooms at workplaces. The rooms used for the audiometric testing were either VIP rooms or the managers' rooms. The background sound pressure levels were monitored by using a precision sound level meter (Rion NL14, Japan) with a type NX-05 octave band filter unit. Sound levels (LAeq) in these testing rooms varied considerably, ranging from 46–74 dBA. For low frequencies (∼500 Hz), the noise levels of most locations ranged from 44–75 dBA, while at higher frequencies (∼8 KHz) the levels ranged from 16–63 dBA.

Participants who were found to have abnormalities (having a hearing threshold >25 dBA at any frequency in either ear) during the screening test were then requested to attend an audiometric diagnostic test. The diagnostic test included the pure tone air conduction hearing thresholds at the following octave frequencies 500, 1,000, 2,000, 3,000, 4,000, 6,000, and 8,000 Hz for both ears. Bone conduction audiometry was performed by using the method described by the British Society of Audiology [2], giving thresholds at octave frequencies of 500, 1,000, 2,000, 3,000, and 4,000 Hz in 5-dBA steps. The masking of the non-testing ear was done as required. If the air conduction thresholds of all the tested frequencies were within 25 dBA, no bone conduction measures were taken.

The audiometric diagnostic test was carried out inside a sound-proof audiometric booth (Nap Acoustic Silentflo Room, Australia) by using a Madsen audiometer (Orbiter 922, Denmark) at the Department of Community and Family Medicine, Lek Yuen Health Centre in Shatin, or at the Hong Kong Federation of Trade Unions Clinic (Booth: Nap Acoustic Silentflo Room, Australia; audiometer: Interacoustics AD 30, Denmark) in Mongkok.

Objective calibrations of all audiometers were carried out once every six months to adjust the sound pressure output in the left and right earphones by using an artificial ear. Biological calibration checks of audiometer functions were carried out before the start of the tests on each day of fieldwork.

### Classification of hearing impairments

The classification of hearing defects used in this study was suggested by Klockhoff *et al.* [13], which classification is often used to evaluate hearing damage for noise-exposed subjects [14]. Briefly, it is based on the results of audiometric tests for each ear at the frequencies of 500, 1,000, 2,000, 3,000, 4,000, 6,000, and 8,000 Hz. The hearing threshold in each ear was classified into five well-defined fields A, B, C, D, and E, depending on the frequency range and the threshold. Fields A and E covered the lower frequency range from 0.5 to 2 KHz, with cut-offs of 30/35 dB at 0.5 KHz and 25/30 dB at 1 and 2 KHz. Field A indicated no clinical impairment in that frequency range. Fields B, C and D covered the higher frequency range from 3 to 6 KHz, with B indicating no clinical impairment in that frequency range. The cut-off between B and C was 25/30 dB and that between C and D was 60/65 dB for 3, 4 and 6 KHz. From the location of the hearing thresholds in these five fields, a “Klockhoff digit” of 1–5 was obtained for each ear. Digit 1 denoted normal hearing (all thresholds within Fields A and B); Digit 2 indicated slight hearing loss in the higher frequencies only, with thresholds of lower frequencies all within Field A and one or more higher frequencies reaching Field C; Digit 3 indicated moderate high tone loss, with thresholds of lower frequencies still within Field A and one or more higher frequencies reaching Field D; Digit 4 denoted severe high tone loss with speech frequencies being affected (higher frequencies mainly in Field D and threshold of one or more lower frequencies reaching Field E). Digits 2, 3 and 4 reflect predominantly high tone hearing loss, which is most commonly a result of noise exposures and/or aging in the study population. Digit 5 included other combinations of fields that might represent low-tone loss or flat loss and suggested hearing damage due to causes other than noises. The audiogram of each subject was then combined into a two-digit number, with the first digit indicating the hearing loss in the right ear and the second in the left ear.

The term ‘Presumptive Noise Induced Hearing Loss’ (P-NIHL) was used when high tone hearing impairment (Digits 2, 3, and 4) was detected in any subject in this study. There are three levels of P-NIHL, namely, slight, moderate, and severe high-tone loss. According to the above coding classification, P-NIHL could be made up of combinations with “12”, “21”, “13”, “31”, “14”, “41”, “22”, “23”, “32”, “24”, “42”, “33”, “34”, “43”, and “44” when the differences in the average hearing levels at 3,000, 4,000, and 6,000 Hz between the better and poorer ear did not exceed 30 dBA [10]. It is usually accepted that occupational hearing loss is bilateral and fairly symmetrical and that asymmetric hearing loss is usually caused by firearms or disease.

To indicate the prevalence of moderate and severe high tone loss in our study population, we used the term “High Tone Loss” (HTL) in this report, which combined P-NIHL coding “33”, “34”, “43”, and “44”.

### Data management and analysis

The double entry of data into computers was done independently by two research assistants and compared subsequently. Any mismatch was corrected after checking the corresponding questionnaire and the test record. Descriptive statistics were used for calculating the means of the continuous variables and the prevalence of the categorical variables. The logistic model was used for calculating the odds ratios. We considered p values of <0.05 to be statistically significant, and 95% confidence intervals were also presented. All data analyses were performed by using the Statistical Package for the Social Science (SPSS for Windows 14.0, SPSS Inc., Chicago, IL).

## Results

The mean age (SD) of the 733 entertainment employees was 38.6 (9.4) years, which was younger than that of the 937 restaurant workers (their mean age was 41.9 (11.7) years). There were more females working in the Chinese restaurants. The mean duration (SD) of working in the entertainment sector was 7.6 (7.1) years, while the service duration for restaurant workers was 10.3 (9.5) years. The general characteristics of the entertainment employees and restaurant workers are presented in Table 1.

**Table 1:** General characteristics of the participants.

Variables	Restaurant workers		Entertainment employees	
	No.	%	No.	%
**Age group (years)**				
<30	153	16.33	141	19.2
30–	223	23.80	253	34.5
40–	335	35.75	240	32.8
50–	226	24.12	99	13.5
**Sex**				
Male	396	42.26	402	54.8
Female	541	57.74	331	45.2
**Education**				
No formal education	58	6.21	51	7
Primary School	339	36.3	191	26
Secondary School	528	56.53	256	35
Post-secondary	9	0.96	235	32
**Sub-sector**				
**For restaurant workers:**				
Service workers	545	58.16	-	-
Dishwashers	81	8.65	-	-
Kitchen workers	247	26.36	-	-
Cooks	64	6.83	-	-
**For entertainment employees:**				
Radio/TV stations	-	-	262	35.7
Halls/auditoria of the LCSD	-	-	438	59.8
Karaoke bars	-	-	33	4.5
**Service duration (years)**				
<5	360	38.5	320	49.8
5–15	323	34.6	214	33.3
15-	251	26.9	108	16.9
**Smoking**				
Never	563	60.09	593	80.9
Past	90	9.61	32	4.4
Current	280	29.88	108	14.7
**Drinking**				
Never	604	64.46	316	43.1
Occasionally	271	28.92	381	52.0
Daily	57	6.08	34	4.6

The main source of noise inside restaurants was from the stoves. Other sources included dishwashing machines and ventilation systems, such as fume extractors. The measurements of environmental noise in different areas are shown in Table 2. Serving areas generally had lower environmental noise levels.

**Table 2:** A-weighted sound pressure levels (LAeq) in different areas of restaurants.

Workplace	No. of	Duration (minutes)		LAeq (dB)			
	measurements	Mean	(SD*)	Mean	(SD*)	Min.	Max.
Cooking	17	3.2	(0.7)	86.9	(5.7)	73	97
Barbecuing	3	3.0	(0)	75.0	(7.6)	67	82
Dim sum steaming	4	3.0	(0)	82.3	(3.6)	79	87
Dishwashing	11	3.0	(0)	82.5	(3.6)	74	86
Service	13	10.9	(15.8)	75.9	(5.6)	66	87

* : Standard deviation.

A total of 22 personal dosimetric measurements were taken from 20 cooks and two dishwashers (Table 3). Higher readings were recorded when the stoves were operating and the workers were washing dishes. The data of the dosimetry of participants with different job titles are given in Table 3. The personal dosimetric measurements showed that the chief cooks were exposed to a mean noise level of 95.4 dB(A) when they were cooking. The results of three samples were >100 dB(A) in the personal daily exposure levels (LEPd). The overall mean LEPd among 20 cooks was 92.9 dB(A). Dishwashers were also exposed to a mean noise level of 90.5 dB(A) (LEPd).

**Table 3:** Personal dosimetry for participants with different job nature.

Job nature	No. of	Mean Duration	LEPd* (dB(A))			
	measurements	(minutes)	Mean	(SD)	Min.	Max.
**Restaurant workers:**						
Chief cooks	11	98.6	95.4	(6.3)	88	108
Second cooks	6	86.5	91.0	(3.0)	86	94
Barbecue cooks	1	170.0	88.0	-	-	-
Dim sum cooks	2	75.0	87.5	(0.7)	87	88
Dishwashers	2	89.0	90.5	(2.1)	89	92
**Entertainment employees:**						
**TV program**						
Filming with headphone (TV)	1	138	101.9	-	-	-
Carpenter (TV)	1	365	91.6	-	-	-
Editing (TV)	1	431	80.0	-	-	-
Filming (TV)	1	375	78.0	-	-	-
Camera (TV)	1	80	74.3	-	-	-
**Halls/auditoria of LCSD**						
Backstage (LCSD)	11	120	86.1	(5.8)	78.4	99.8
Frontstage (LCSD)	15	209	82.3	(6.6)	71.5	96.2
**Karaoke bar**						
Karaoke disc jockeys	1	442	82.6	-	-	-
Karaoke servicing staff	1	507	81.9	-	-	-

A total of 34 personal dosimetric measurements of typical entertainment employees who did not wear headsets were taken from various job groups. Noise levels ranged from 74.3 to 101.9 dBA. Filming with headphone (TV) and Carpenter (TV) had the highest exposure to mean noise levels. The results of dosimetric measurements for the entertainment employees are also presented in Table 3.

The noise levels of the radio program presenters who wore headsets are presented in Table 4. The noise levels ranged from 89.6 to 110.1 dBA. These employees generally had higher exposure levels compared to other entertainment employees and restaurant workers.

**Table 4:** Noise measurements for radio programs presenters wearing headset in the radio and television station.

Program type	No. of measurements	Mean Duration	LAeq* (dB)			
		(minutes)	Mean	(SD)	Min.	Max.
Classical music	11	106	89.6	9.0	66.0	100.9
Music drama	1	180	97.8	-	-	-
Pop music	8	131	101.3	4.8	91.8	108.3
Entertainment talk show	4	120	102.1	2.7	98.5	105.0
Variety magazine	2	210	104.8	0.2	104.6	104.9
Phone-in	2	60	110.1	1.6	108.9	111.2

* The equivalent continuous A-weighted sound pressure level.

**Figure 1:**
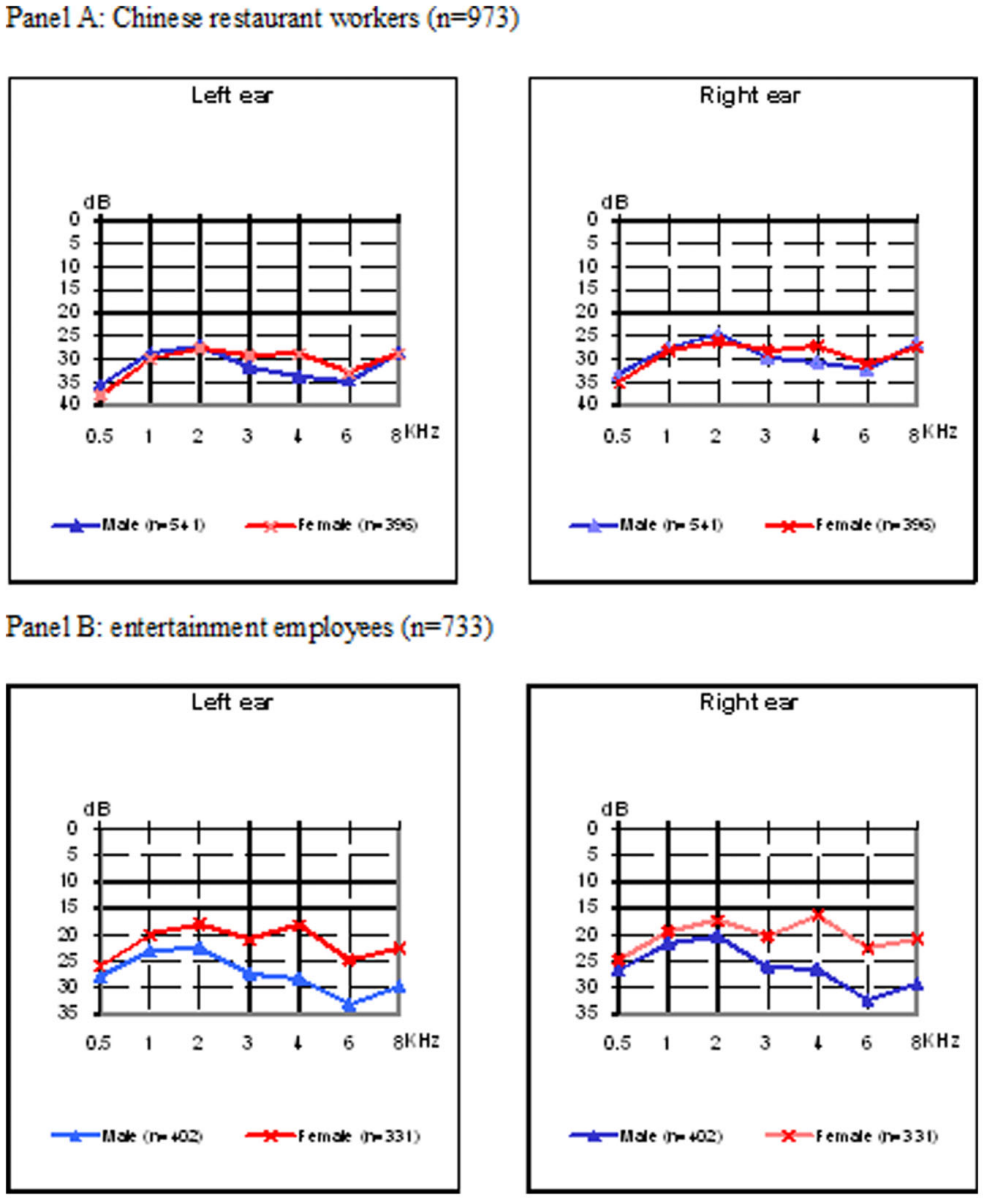
Mean hearing thresholds by gender among participants. Panel A: Chinese restaurant workers (n = 973). Panel B: entertainment employees (n = 733).

The mean hearing thresholds by frequency for restaurant workers stratified by gender are shown in Panel A in Figure 1. The pattern of hearing thresholds shows a dip at 3, 4 and 6 KHz, characterizing of the “noise-induced hearing loss” pattern. Men had worse thresholds at 3, 4 and 6 KHz than women. The mean hearing thresholds by frequency of entrainment employees stratified by gender are shown in Panel B in Figure 1. The pattern of hearing threshold shows a dip at 6 kHz, which is also the characteristic of the “noise-induced hearing loss” pattern. Men had worse thresholds than women, especially at the frequency of 3 KHz or above.

The prevalence of P-NIHL and HTL, were 23.7% (95% CI: 21.0–26.4) and 11.3% (95% CI: 9.3–13.3) among restaurant workers, respectively. For entertainment employees, the corresponding figures were 38.6% (95% CI: 35.1–42.1%) and 7.0% (95% CI: 5.2–8.8%). Table 5 shows the odds ratios and prevalence of P-NIHL in the participants stratified by risk factors among restaurant workers. Being male, older, and daily alcohol consumption were associated with noise-induced hearing impairment. In addition, kitchen worker and cook were associated with lower prevalence of NIHL. Table 6 shows the odds ratios and prevalence of P-NIHL in the participants stratified by risk factors among the entertainment employees.

**Table 5:** Stratified prevalence and odds ratios of presumptive noise-induced hearing loss (P-NIHL) among the restaurant workers in Hong Kong.

	Restaurant workers (n = 937)				
	All	Cases	Prevalence (%)	Crude OR (95%CI)	Adjusted OR* (95%CI)
**Gender**					
Female	541	78	19.7	Reference	Reference
Male	396	144	26.3	3.47 (2.53, 4.75)	2.17 (1.40, 3.36)
**Age group (years)**					
<30	153	14	9.2	Reference	Reference
30–39	223	55	24.7	3.25 (1.73, 6.09)	2.36 (1.31, 4.24)
40–49	335	96	28.7	3.99 (2.19, 7.25)	3.08 (1.72, 5.49)
≥50	226	57	25.2	3.35 (1.79, 6.26)	2.79 (1.50, 5.18)
**Service duration (years)**					
<5	360	71	19.7	Reference	Reference
5–15	323	74	22.9	1.21 (0.84, 1.75)	0.90 (0.62, 1.31)
15	251	77	30.7	1.80 (1.24, 2.62)	1.00 (0.65, 1.56)
**Job group**					
Service worker	545	141	25.9	Reference	Reference
Dishwasher		18	22.2	0.75 (0.44, 1.28)	0.92 (0.52, 1.63)
Kitchen worker	247	52	21.1	0.80 (0.57, 1.12)	0.59 (0.40, 0.88)
Cook	64	11	17.2	0.59 (0.31, 1.13)	0.42 (0.21, 0.83)
**Smoking**					
Never	563	126	22.4	Reference	Reference
Past	90	28	31.1	1.57 (0.96, 2.55)	1.08 (0.64, 1.83)
Current	280	68	24.3	1.11 (0.79, 1.56)	1.03 (0.70, 1.52)
**Drinking**					
Never	604	135	22.4	Reference	Reference
Occasionally	271	63	23.2	1.05 (0.75, 1.48)	0.95 (0.67, 1.36)
Daily	57	23	40.4	2.35 (1.34, 4.13)	1.78 (1.00, 3.19)

* Adjusted for gender, age, service duration, smoking and drinking.

**Table 6:** Stratified prevalence and odds ratios of presumptive noise-induced hearing loss (P-NIHL) among the entertainment employees in Hong Kong.

	Entertainment employees (n = 733)			
	All	Cases	Prevalence (%)	OR (95%CI)
**Gender**				
Female	331	98	29.6	Reference
Male	402	185	46.0	2.03 (1.49, 2.75)
**Age group (years)**				
<30	141	28	19.9	Reference
30–39	253	92	36.3	2.31 (1.42, 3.75)
40–49	240	113	47.1	3.59 (2.21, 5.83)
≥50	99	50	50.5	4.12 (2.33, 7.29)
**Service duration (years)**				
<5	320	67	23.9	Reference
5–15	214	83	31.3	2.39 (1.63, 3.52)
>15	108	55	43.6	3.92 (2.47, 6.23)
**Smoking**				
Never	593	175	29.5	Reference
Past	32	16	50.0	2.39 (1.17, 4.88)
Current	108	42	38.9	1.52 (0.98, 2.33)
**Drinking**				
Never	316	91	28.8	Reference
Occasionally	381	118	31.0	1.11 (0.80, 1.54)
Daily	34	20	60.0	3.53 (1.71, 7.29)

## Discussion

Our survey examined noise exposures and hearing impairment in Chinese restaurant workers and entertainment employees in Hong Kong. Our results show that noise exposure is common and excessive in Chinese restaurants and in the entertainment industry in Hong Kong. From our representative sample of Chinese restaurant workers we estimated that about 47% of workers in Chinese restaurants are exposed to noise levels of above 85 dB(A). The mean hearing thresholds show a typical dip at 3 to 6 KHz and a substantial proportion of restaurant workers fulfilled the criteria for P-NIHL and HTL (23.7% suffer from P-NIHK and 11.3% suffer from HTL) [15]; for the entertainment employees, our results show that excessive sound exposure was common for radio program presenters who wore a headset during work in the radio and television stations. Many of them were exposed to a mean sound level >100 dBA. For those filming with headphones and Carpenters working for TV programs in the radio and television station, they had sound exposures >85 dBA; for cultural performance halls or the auditoria of the LCSD, the employees working backstage had a higher mean sound exposure level than those working frontstage (86.1 dBA vs 82.3 dBA). Although in our data, the employees who worked in karaoke bars were not exposed to excessive noise levels (<85 dBA), we should be very cautious because the response rate for karaoke bars was low (33.3%) and the number of measurements was small (only two participants were measured). Our survey suggests that a high level of sound exposure is common and that NIHL is an important public health problem in Chinese restaurant workers and entertainment employees in Hong Kong, and that the situation is more severe in entertainment employees, and it is especially severe for those program presenters who wear a headset when working in the radio and television stations.

Regarding the noise exposures and prevalence of hearing loss in the manufacturing industry in Hong Kong, previous studies have shown that 38% of workers were exposed to noise levels of 90 dB(A) or more, and that 19% suffered from HTL, which was higher than the HTL prevalence (7.0%) in our entertainment employees and the HTL prevalence (11.3%) in Chinese restaurant workers, but there are no data on P-NIHL in this study [13]. Another study which focused on the transport industry showed that 43.2% of the workers were exposed to noise levels of 85–89 dBA, and 32.5% were exposed to over 90 dB(A). The prevalence of P-NIHL and HTL in the transport industry was 32.3% and 8.4%, respectively [16]. When stratified by sex, there was higher prevalence of NIHL in restaurant female workers (19.7%) than that in transportation female workers (10.4%), but the prevalence in restaurant male workers (26.3%) was lower than that in transportation workers (26.3%). When stratified by age group, the prevalence of NIHL in transportation workers were much higher especially in older age group (46.8% in 40–49 years group and 57.5% in 50–59 years group). In short, compared to the traditional manufacturing industries in Hong Kong, there is a higher proportion of Chinese restaurant workers who are exposed to excessive noise, but a lower prevalence of NIHL among the workers. Although the sample of entertainment employees was not a representative sample in our study, the results show that the prevalence of P-NIHL and HTL in entertainment employees was similar to those in the transport industry [10].

There is a little information about NIHL for restaurants in other countries. The study conducted by Lebo *et al.* in San Francisco showed that the noise levels generally ranged from 60–80 dB(A), with the peak at 87 dB(A) [17]. In this survey, the mean noise level in the service area was 75.9 dB(A) (ranging from 66–87 dB(A)), which was similar to that in Lebo's study. However, noise levels in kitchens were much higher (mean noise level was 86.9 dB(A) during cooking). This was expected because Chinese-style cooking requires high temperatures and pressurized gas stoves which generate a lot of noise. Our survey shows that pressurized gas stoves are the major source of noise in the kitchens of Chinese restaurants in Hong Kong.

Hitherto, there has been limited information on hearing loss on employees in the entertainment industries worldwide. Some overseas studies for employees working at music venues showed that the prevalence generally ranged from 29–50%. However, the sample sizes of these studies were small with there being 14–82 participants in each study [7]–[9]. In our study we found that the sound levels were extremely high for those employees wearing headsets in the radio and television stations. Most of them had an exposure of >91 dBA, with the maximum up to 111.2 dBA. This is possibly because the employees had to increase the sound level in an attempt to drown outside noise [18]. Hodgetts *et al.* reported that environmental noises and headphone styles were associated with increased preferred listening levels among the adults using MP3 players [19]. There is little information to show the sound exposure levels for professional employees who wear headsets during work in entertainment venues, but a number of studies on people using personal music players (PMPs) for leisure music have shown that the output of PMPs could attain 110 dBA, with the average sound level exceeding 85 dBA [6]. This is in conformity with our results for the employees who wear headsets during work. Prolonged and excessive sound exposure through PMPs was associated with the risk of developing hearing loss [6]. The sound levels from speakers are generally lower than those from earphones. Students who listen to music using speakers were found to have significantly lower hearing thresholds than those using PMPs [20].

Aging has been well documented as an important risk factor for hearing loss. However, presbycusis usually shows a pattern on an audiogram different from that of NIHL. Generally presbycusis shows the greatest threshold shifts at higher frequencies, leading to a downward sloping curve without a “notch” [3], [21]. In our survey, the audiogram shows a typical “notch” at 3,000–6,000 Hz for both ears (Fig. 1), and this could not be entirely attributed to the age effect [3], [15]. Furthermore, the “Klockhoff digit” method was used to classify hearing loss and losses at digit 5 were excluded. This enabled us to exclude the serious hearing damages which results from other causes [13].

Our results show that being male, and older were significantly associated with an increased risk of hearing impairment in both restaurant workers and entertainment employees. These findings are in conformity with previous studies [5], [22]–[24]. Our survey also showed that daily consumption of alcohol was associated with increased risk of hearing impairment. Some previous studies suggested that mild drinking had a protective effect [24], but that frequent alcohol consumption increased the risk [25]. The effect of smoking on hearing loss is still controversial. Rosenhall *et al.* reported an association between hearing levels and smoking [25]. However, no association was found in the Framingham study [26]. Another two studies found a dosage effect of smoking on hearing loss [27], [28], and an interaction between smoking and occupational noise was also reported [29], [30]. We need to point out that our results show that kitchen worker and cook, who are generally with higher exposure level, were associated with lower risk of NIHL. This is possibly because of the cross-sectional study design. The cook and kitchen worker with NIHL already quit their job because of the disease. Health worker effect might also be another reason for this phenomenon.

There are several disadvantages in our study which limited us for thoroughly assessing the relationship of NIHL with noise exposures: (1) due to the shortage of resources, information on many potential risk factors could not be collected, and we explored only the associations between NIHL and some demographic factors as well as smoking and drinking. (2) the cross-sectional nature did not allow us to assess any temporal relationship of NIHL with noise level as well as the potential risk factors. Prospective cohort studies are required to thoroughly assess the relationship between sound exposures and NIHL, as well as the effects of other risk factors/confounders on the relationship; and (3) the current measurements of noise exposure levels might not accurately reflect past exposures. Other limitations in our survey include the low response rate for karaoke bars, the non-representative sampling in the entertainment industry, and small sample size of each sub-sector in the entertainment industry. However, because of the wide scope and various job natures in the entertainment industry, a representative sample is difficult to obtain unless a census can be carried out for the whole entertainment industry.

Our results have important public health implications. NIHL is permanent and irreversible, but actually it is completely preventable [4]. Our results show that exposure to high sound levels is common for Chinese restaurant workers and entertainment employees in Hong Kong, particularly for cooks and dishwashers in restaurants, and for those radio program presenters who wear headsets in stations; this is because many of them are exposed to a sound level of >90 dBA. In Hong Kong, it is estimated that there are around 120,000 catering workers in Chinese restaurants [31]. Based on our results, a total of 56,400 (47%) workers are being exposed to noise levels of 85 dB(A) or above. Around 28,000 (23.7%) workers are suffering from P-NIHL and 13,000 (11.3%) are suffering from HTL. The situation for entertainment employees is worse than restaurant workers; this is because the entertainment employees are exposed to higher levels of noise and have higher incidence of hearing loss. Emergency strategies should be taken to prevent NIHL. Engineering controls should be considered, and they should be especially targeted at noisy pressurized gas stoves. Comprehensive hearing conservation programs, such as noise assessments, audiometric monitoring of workers' hearing, appropriate use of hearing protectors, education for workers, record-keeping, and also program evaluation, should be introduced in the Chinese restaurant industry. In addition, the Labor Department of Hong Kong is working towards the introduction of legislation which requires periodic auditory examinations of workers who are exposed to excessive noise in establishments under the Factories and Industrial Undertakings Ordinance. The successful enactment of this law will be an important milestone in the control of NIHL in Hong Kong. Our survey provides a solid basis for extending the future legislative coverage to workplaces outside the industrial undertakings.
